# Prospective Analysis of Risk for Hypothyroidism after Hemithyroidectomy

**DOI:** 10.1155/2015/313971

**Published:** 2015-03-30

**Authors:** Virgilijus Beisa, Darius Kazanavicius, Arminas Skrebunas, Gintaras Simutis, Justinas Ivaska, Kestutis Strupas

**Affiliations:** ^1^Clinic of Gastroenterology, Nephrourology and Surgery, Center of Abdominal Surgery, Faculty of Medicine, Vilnius University, Santariskiu 2, LT-08661 Vilnius, Lithuania; ^2^Clinic of Ear, Nose, Throat and Eye Diseases, Center of Ear, Nose and Throat Diseases, Faculty of Medicine, Vilnius University, Santariskiu 2, LT-08661 Vilnius, Lithuania

## Abstract

*Objectives*. To evaluate risk factors and to develop a simple scoring system to grade the risk of postoperative hypothyroidism (PH).* Methods*. In a controlled prospective study, 109 patients, who underwent hemithyroidectomy for a benign thyroid disease, were followed up for 12 months. The relation between clinical data and PH was analyzed for significance. A risk scoring system based on significant risk factors and clinical implications was developed.* Results*. The significant risk factors of PH were higher TSH (thyroid-stimulating hormone) level and lower ratio of the remaining thyroid weight to the patient's weight (derived weight index). Based on the log of risk factor, preoperative TSH level greater than 1.4 mU/L was assigned 2 points; 1 point was for 0.8–1.4 mU/L. The derived weight index lower than 0.8 g/kg was assigned 1 point. A risk scoring system was calculated by summing the scores. The incidences of PH were 7.3%, 30.4%, and 69.2% according to the risk scores of 0-1, 2, and 3.* Conclusion*. Risk factors for PH are higher preoperative TSH level and lower derived weight index. Our developed risk scoring system is a valid and reliable tool to identify patients who are at risk for PH before surgery.

## 1. Introduction

Postoperative hypothyroidism (PH) after hemithyroidectomy remains unpredictable [1, 2]. The reported incidence ranges from 0% to 43% [3]. Early symptoms of PH can be sleepiness, fatigue, and weight gain. PH is treated with L-thyroxine, which can also lead to arrhythmias, osteopenia, and osteoporosis [4]. However, some patients after hemithyroidectomy will not require thyroid hormone replacement because they remain in the euthyroid status. How could we predict thyroid function after hemithyroidectomy?

Ability to preoperatively recognize who are most at risk for developing PH would help to choose an optimal volume of thyroid resection. While there is a general consensus that the surgical treatment of thyroid cancers is total thyroidectomy, the optimal operative strategy for patients with benign thyroid disease remains controversial. Current indications for hemithyroidectomy are large benign nodules, follicular neoplasm, and compression-induced symptoms [5, 6]. The main reason for performing hemithyroidectomy is presumed lower incidence of postoperative complications, including recurrent laryngeal nerve palsy and hypoparathyroidism, and an attempt to achieve postoperative euthyroid status [7]. However, if there is a small (<1 cm) nodule in the remaining gland but the patient is at high risk of developing PH, the optimal type of operation could be total thyroidectomy. Moreover, prediction of hypothyroidism would improve patient care: closer monitoring or earlier initiation of thyroid hormone replacement therapy could be advisable for the high-risk patients.

This issue has led to our interest in establishing precise and simple criteria for predicting residual thyroid function preoperatively. Recently, our retrospective pilot study has identified three risk factors for the PH after hemithyroidectomy: preoperative TSH level, age, and ratio of the remaining thyroid weight to the patient's weight [8]. Our current aim was to perform a prospective study with expanded list of potential risk factors and to develop a scoring system to grade the risk of PH.

## 2. Materials and Methods

We performed a prospective study of patients undergoing hemithyroidectomy from January 2010 to December 2012. Hemithyroidectomy was defined as the removal of the unilateral thyroid lobe, isthmus, and, where present, the pyramidal lobe of the thyroid. Patients were excluded from the study if (1) they were preoperatively on thyroid hormone for preexisting hypothyroidism or/and to prevent nodule growth; (2) there was pathologic diagnosis of thyroid malignancy; (3) they later underwent total thyroidectomy; (4) they were on medications known to alter thyroid hormone or serum TSH level. All patients were in euthyroid state preoperatively.

Patients were divided into two groups according to the thyroid function after hemithyroidectomy: euthyroid group, patients with normal thyroid function, TSH, and levothyroxine (LT3) and liothyronine (LT4) levels within normal limits; hypothyroid group, patients with hypothyroidism. Hypothyroidism was defined as an increased TSH level with or without subnormal thyroid hormone levels. A normal range for TSH in our institution was from 0.4 to 4.0 mU/L. Postoperative TSH and LT3 and LT4 tests were performed 2, 6, and 12 months after the surgery.

Patients were analyzed for possible risk factors as gender, age, body weight, height, BMI (body mass index), preoperative serum TSH, free T4, T3 hormone levels, antithyroid peroxidase level, thyroid glands characteristics, and extra and final pathologic analysis.

The weight (*M*) of the remnant lobe was calculated using the measurements from ultrasonography in the following equation according to the ellipsoid method [19]: *M* (g) = 0.508 × (lobe length (cm)) × (lobe width (cm)) × (lobe depth (cm)). We calculated ratio (*A*) of the remaining thyroid weight to the patient's weight: *A* (g/kg) = *M* (g)/patient's body weight (kg). This ratio was defined as derived weight index.

The statistical significance of the difference between euthyroid and hypothyroid groups was analyzed using Student's *t*-test for continuous variables and the chi-squared test for nominal variables. Logistic multiple regression was then performed using all factors found significant on univariate analysis. Based on the log (odds ratio) of each risk factor, factors were assigned to a score based on its value to hypothyroidism. The scoring system was verified with the Hosmer-Lemeshow goodness-of-fit test. Values of *P* < 0.05 were considered statistically significant.

## 3. Results

**Figure 1:**
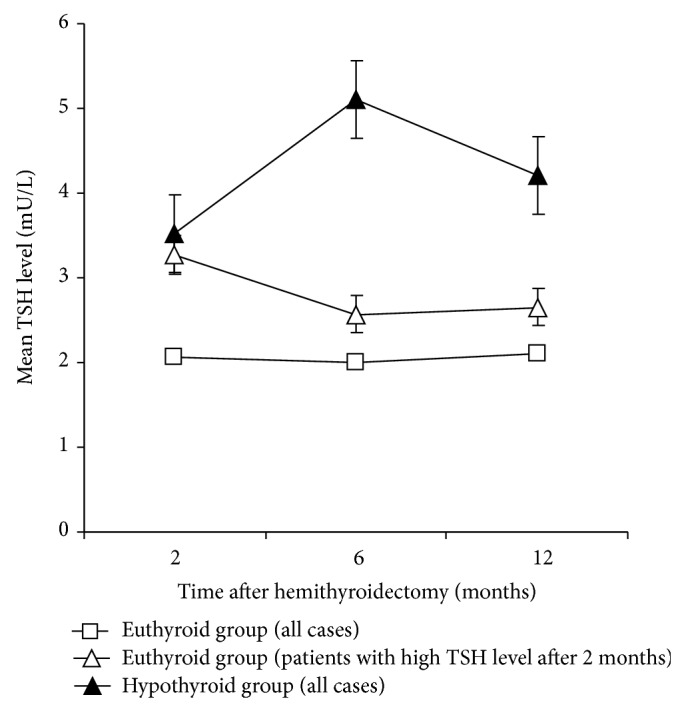
Posthemithyroidectomy TSH level dynamics of euthyroid and hypothyroid patients.

PH developed in 20 (18.3%) out of 109 patients. 90% of these cases manifest in 2–6 months after the operation: hypothyroidism was diagnosed after 2 and 6 months after the surgery in 12 (60.0%) and 6 (30.0%) patients, respectively. 2 (10.0%) new cases of hypothyroidism were documented after 12 months postoperatively. The remaining patients remained euthyroid throughout the study. We have taken notice of the patients who had high but normal postoperative serum TSH level after 2 months and did not take thyroid hormone postoperatively. Serum TSH level of those patients was decreasing progressively 0.7 mU/L per 10 months (Figure 1). TSH level dynamics of these patients compared with all euthyroid and hypothyroid patients after 2, 6, and 12 months are presented in Figure 1. Characteristics of patients in euthyroid and hypothyroid groups are presented in Table 1.

The mean preoperative serum TSH level was 0.85  ±  0.46 mU/L in euthyroid group, compared with 1.42 ± 0.67 mU/L in hypothyroid group (*P* = 0.00004). The mean remaining thyroid weight was 7.05 ± 4.32 g in euthyroid group and 4.17 ± 1.82 g in hypothyroid group (*P* = 0.005) and the derived weight index (the ratio of the remaining thyroid weight to the patient's weight) was 0.094 ± 0.050 g/kg in euthyroid group and 0.057 ± 0.025 g/kg in hypothyroid group (*P* = 0.001).

Interestingly, the mean patients height in euthyroid group was 1.70 ± 0.09 m compared to 1.66 ± 0.08 m in hypothyroid group (*P* = 0.054). However, the groups did not establish a significant difference between patients' weight (73.46 ± 15.46 kg compared with 74.70 ± 16.56 kg (*P* = 0.77)) and BMI (25.19 ± 4.24 versus 27.10 ± 6.08 (*P* = 0.11)). Also, there was no significant difference between patients' age, gender, preoperative serum antithyroid peroxidase, free T4, T3 level, right versus left hemithyroidectomy, thyroid echogenicity, nodules' count, and weight in the resected and remaining gland or pathologic analysis (Table 2). The most common final pathologic analysis demonstrated follicular adenomas (37.6%) and multinodular goiters (32.1%).

Significant predictors of PH in the multivariate analysis included the preoperative serum TSH level (*P* = 0.002) and the derived weight index (*P* = 0.009). These predictors were used in the logistic model to predict PH. According to the logistic regression equation, the prediction model was simplified to cutoff values of each variable. Variable of the preoperative TSH level was divided into three groups (cutoff values 0.8 and 1.4 mU/L) and the derived weight index was divided into two groups (cutoff value 0.08 g/kg). Considering the probability of the PH development in each variable's group we assigned the highest score of 2 to preoperative TSH level and the highest score of 1 was assigned to the derived weight index (Table 3). The sum of scores was estimated. The incidences of hypothyroidism were 7.3%, 30.4%, and 69.2% according to the risk scores of 0-1, 2, and 3, respectively (Table 4). The Hosmer-Lemeshow goodness-of-fit test suggested that the model was well calibrated (*P* = 0.69). Overall predictive capacity of the model to predict PH is 85.4%.

## 4. Discussion

Our finding that 18.3% of patients who developed hypothyroidism after undergoing hemithyroidectomy for benign thyroid disease are consistent with others reported in the literature. The reported incidence ranges from 0% to 43%, with most between 15 and 30% [3]. In our study, to monitor TSH level after hemithyroidectomy serum TSH was assessed at the first 2 months and again at 6 and 12 months postoperatively. It is necessary to wait for at least four to five half-lives of TSH before measuring a serum TSH level postoperatively to get an accurate assessment of the thyroid hormone being produced by the residual thyroid lobe because serum TSH has a half-life of about 7 days [20].

However, 90% of PH patients were detected in 2 and 6 months postoperatively. After 12 months it developed in 10% cases. Serum TSH level of patients who had high but normal postoperative serum TSH level after 2 months was decreasing progressively leading to a normal thyroid function. These patients remained euthyroid throughout the study. Therefore, the state of the final thyroid function could be determined in 12 months after hemithyroidectomy.

We have found that the incidence of PH significantly correlated with higher preoperative serum TSH levels. In addition to the preoperative TSH level, patients who developed PH had lower ratio of the remaining thyroid weight to the patient's weight compared to those who remained in the euthyroid state (*P* = 0.001). Several studies have looked into the risk factors of developing PH (Table 5) [1, 2, 8–18, 21]. They noted that some of the proposed risk factors can be determined only after surgery, whereas others can be detected during the preoperative period. Among them, the preoperative TSH level was commonly noted to have a significant relation with PH. To our knowledge, our studies are the first that show the relation between hypothyroidism and the ratio of the remaining thyroid weight to the patient's weight. This ratio is more sensitive parameter than just the remaining thyroid weight (*P* = 0.005).

Other studies have pointed that thyroiditis is also associated with PH. Patients diagnosed with thyroiditis were found to be more likely to develop hypothyroidism after hemithyroidectomy and the need for thyroid hormone supplementation following hemithyroidectomy was significantly increased [4]. Thyroiditis is also characterized by increased lymphocytic infiltration. It is also stated that lymphocytic infiltration within the thyroid gland, at the time of surgery, is a possible predictor of hypothyroidism [1, 2, 10, 12, 14, 18]. However, these factors can be reliably assessed after the operation and are not suitable for thyroid function prediction before the operation. Therefore, we did not factor this variable in our analysis. Other reports about risk factors for hypothyroidism have also indicated links to anti-TPO antibodies, multinodular goiter, and preoperative thyrotoxicosis. However, we did not find any significant difference between our two groups with respect to age, gender, the patient's weight, body mass index, anti-TPO antibodies, thyroid echogenicity, nodules' count, and weight in the resected and remaining gland or pathologic analysis.

There are some limitations that should be taken into account when interpreting this study. The weight of the remnant lobe was calculated using the measurements from ultrasonography which is dependent on a physician. Also, though the technical performance of the procedure of hemithyroidectomy is quite straightforward and is supposed to include resection of the isthmus, we cannot exclude that small variations in the extent of the resection may exist and may impact the risk of hypothyroidism because smaller remnant thyroid volume has been shown to increase the risk of PH.

However, our data was efficient to create a simple and accurate risk scoring system to predict hypothyroidism before surgery. From a practical viewpoint, being able to predict the individual patient's probability of developing PH is of value when making diagnostic and therapeutic plans, choosing a type of operation. Closer monitoring or earlier initiation of thyroid hormone replacement therapy should be advisable for the high-risk patients.

## 5. Conclusions

After hemithyroidectomy, approximately 1 in 5 patients experience hypothyroidism. 90% of these cases manifest in 2–6 months after the operation and the final state of thyroid function could be determined in 12 months after hemithyroidectomy. Current study shows that the most important predictors in developing hypothyroidism are preoperative serum TSH level and the ratio of the remaining thyroid weight to the patient's weight. Simple risk scoring system proposed in our study is a valid and reliable tool to identify patients who are at risk for posthemithyroidectomy hypothyroidism before surgery.

## Figures and Tables

**Table 1:** Characteristics of patients in the euthyroid group and hypothyroid group.

Variable	Euthyroid group	Hypothyroid group	*P*
Age, years	42.3 ± 11.8	44.2 ± 17.8	*0.58 *
Male	16.9%	15.0%	0.27
Female	83.1%	85.0%	
Patients height, m	1.70 ± 0.09	1.66 ± 0.08	0.05
Patients weight, kg	73.5 ± 15.5	74.7 ± 16.6	0.77
Patients BMI	25.2 ± 4.2	27.1 ± 6.1	*0.11 *
ASA ≤ 2	64.2%	61.8%	*0.10 *

BMI: body mass index; n.s.: difference being not significant.

**Table 2:** Univariate analysis between posthemithyroidectomy euthyroid and hypothyroid patients.

Predictor	Euthyroid group	Hypothyroid group	*P*
Age, years	42.3 ± 11.8	44.2 ± 17.8	0.58
Patients height, m	1.70 ± 0.09	1.66 ± 0.08	0.05
Patients BMI	25.2 ± 4.2	27.1 ± 6.1	*0.11 *
Nodule number			*0.15 *
Single, %	69.1	67.6	
Multiple, %	30.9	32.4	
Remaining thyroid weight, g	7.05 ± 4.32	4.17 ± 1.82	*0.005 *
Derived weight index, g/kg	0.094 ± 0.050	0.057 ± 0.025	*0.001 *
Preoperative serum TSH level, mU/L	0.85 ± 0.46	1.42 ± 0.67	*0.00004 *
Preoperative serum LT3 level, mU/L	4.4 ± 0.9	4.1 ± 0.7	0.07
Preoperative serum LT4 level, mU/L	13.3 ± 2.2	12.5 ± 3.3	0.28
Preoperative ATPO level	30.7 ± 238.8	9.0 ± 16.3	0.45
Side of hemithyroidectomy, right, %	54.3	75.0	0.1

TSH: thyroid-stimulating hormone; ATPO: antithyroid peroxidase; BMI: body mass index; n.s.: difference being not significant.

**Table 3:** Prediction scores for each significant factor to produce the scoring system.

Factor	Criteria	Score
Preoperative TSH level, mU/L	≤0.8	0
	0.9–1.4	1
	>1.4	2

Derived weight index, g/kg	≥0.08	0
	<0.08	1

**Table 4:** Probability of postoperative hypothyroidism according to the risk score.

Cutoff point	Sensitivity (%)	Specificity (%)	Probability of hypothyroidism (%)
0	96	74	**3.5**
1	92	64	**8.0**
2	75	82	**30.4**
3	38	87	**69.2**

**Table 5:** Studies regarding the predictors of PH.

Study	Country	Year	*n*	PH incidence	Predictors of hypothyroidism
McHenry and Slusarczyk [2]	USA	2000	71	35%	Lymphocytic infiltration; weight of resected gland
Buchanan and Lee [1]	UK	2001	158	24.1%	Lymphocytic infiltration; presence of thyroid antibody
Miller et al. [9]	USA	2006	90	27%	Preoperative serum TSH level; age
Koh et al. [10]	South Korea	2008	136	42.6%	Preoperative serum TSH level; lymphocytic infiltration; preoperative microsomal antibody; higher thyroglobulin antibody level
Moon et al. [11]	South Korea	2008	132	36.6%	Preoperative serum TSH level; remnant thyroid volume
Wormald et al. [12]	Ireland	2008	82	18.3%	Preoperative serum TSH level; lymphocytic infiltration
de Carlucci Jr. et al. [13]	Brazil	2008	168	32.8%	Preoperative serum TSH level; remnant thyroid volume; higher thyroperoxidase antibody level; right versus left lobectomy
Su et al. [14]	Australia	2009	294	10.9%	Preoperative serum TSH level; thyroiditis; higher thyroid antibodies levels
Beiša et al. [8]	Lithuania	2011	216	22%	Preoperative serum TSH level; age; derived weight index
Tomoda et al. [15]	Japan	2011	260	24.4%	Preoperative serum TSH level; age
Johner et al. [16]	Canada	2011	117	21.6%	Preoperative serum TSH level; lymphocytic infiltration
Chu and Lang [17]	China	2012	263	14.4%	Age; preoperative serum TSH level; longer follow-up; thyroiditis; positive antimicrosomal antibodies
Said et al. [18]	USA	2013	1240	34%	Preoperative serum TSH level; age; thyroiditis
This study	Lithuania	2014	109	18.3%	Preoperative serum TSH level Derived weight index

*n*: number of hemithyroidectomies; TSH: thyroid-stimulating hormone.

## References

[1] Buchanan M. A., Lee D. (2001). Thyroid auto-antibodies, lymphocytic infiltration and the development of post-operative hypothyroidism following hemithyroidectomy for non-toxic nodular goitre. *Journal of the Royal College of Surgeons of Edinburgh*.

[2] McHenry C. R., Slusarczyk S. J. (2000). Hypothyroidisim following hemi-thyroidectomy: incidence, risk factors, and management. *Surgery*.

[3] Verloop H., Louwerens M., Schoones J. W., Kievit J., Smit J. W. A., Dekkers O. M. (2012). Risk of hypothyroidism following hemithyroidectomy: systematic review and meta-analysis of prognostic studies. *Journal of Clinical Endocrinology and Metabolism*.

[4] Stoll S. J., Pitt S. C., Liu J., Schaefer S., Sippel R. S., Chen H. (2009). Thyroid hormone replacement after thyroid lobectomy. *Surgery*.

[5] DeGroot L. J. (2001). Treatment of multinodular goiter by surgery. *Journal of Endocrinological Investigation*.

[6] Cohen-Kerem R., Schachter P., Sheinfeld M., Baron E., Cohen O. (2000). Multinodular goiter: the surgical procedure of choice. *Otolaryngology—Head and Neck Surgery*.

[7] Foster R. S. (1978). Morbidity and mortality after thyroidectomy. *Surgery Gynecology and Obstetrics*.

[8] Beiša V., Kazanavièius D., Skrebunas A., Simutis G., Šileikis A., Strupas K. (2011). Prognosis of thyroid function after hemithyroidectomya. *Central European Journal of Medicine*.

[9] Miller F. R., Paulson D., Prihoda T. J., Otto R. A. (2006). Risk factors for the development of hypothyroidism after hemithyroidectomy. *Archives of Otolaryngology—Head and Neck Surgery*.

[10] Koh Y. W., Lee S. W., Choi E. C. (2008). Prediction of hypothyroidism after hemithyroidectomy: a biochemical and pathological analysis. *European Archives of Oto-Rhino-Laryngology*.

[11] Moon H.-G., Jung E.-J., Park S.-T. (2008). Thyrotropin level and thyroid volume for prediction of hypothyroidism following hemithyroidectomy in an Asian patient cohort. *World Journal of Surgery*.

[12] Wormald R., Sheahan P., Rowley S., Rizkalla H., Toner M., Timon C. (2008). Hemithyroidectomy for benign thyroid disease: who needs follow-up for hypothyroidism?. *Clinical Otolaryngology*.

[13] de Carlucci D., Tavares M. R., Obara M. T., Martins L. A. L., Hojaij F. C., Cernea C. R. (2008). Thyroid function after unilateral total lobectomy: risk factors for postoperative hypothyroidism. *Archives of Otolaryngology: Head and Neck Surgery*.

[14] Su S. Y., Grodski S., Serpell J. W. (2009). Hypothyroidism following hemithyroidectomy: a retrospective review. *Annals of Surgery*.

[15] Tomoda C., Ito Y., Kobayashi K., Miya A., Miyauchi A. (2011). Subclinical hypothyroidism following hemithyroidectomy: a simple risk-scoring system using age and preoperative thyrotropin level. *ORL*.

[16] Johner A., Griffith O. L., Walker B. (2011). Detection and management of hypothyroidism following thyroid lobectomy: evaluation of a clinical algorithm. *Annals of Surgical Oncology*.

[17] Chu K. K.-W., Lang B. H.-H. (2012). Clinicopathologic predictors for early and late biochemical hypothyroidism after hemithyroidectomy. *The American Journal of Surgery*.

[18] Said M., Chiu V., Haigh P. I. (2013). Hypothyroidism after hemithyroidectomy. *World Journal of Surgery*.

[19] Zeromskas P. (2000). *Skydliaukės dydžio ir jos likučio po operacijos nustatymas [Daktaro disertacija]*.

[20] Lombardi G., Panza N., Lupoli G., Leonello D., Carlino M., Minozzi M. (1983). Study of the pituitary-thyroid axis in euthyroid goiter after partial thyroidectomy. *Journal of Endocrinological Investigation*.

[21] Beiša V., Sileikis A., Eismontas V., Strupas K. (2012). Video-assisted loboisthmectomy by the subclavicular approach. A case report. *Wideochirurgia i Inne Techniki Małoinwazyjne*.

